# Sleep Quality and Interoception Are Associated with Generalized Anxiety in Baccalaureate Nursing Students: A Cross-Sectional Study

**DOI:** 10.3390/nursrep14020090

**Published:** 2024-05-13

**Authors:** Laura A. Robinson, Pamela R. Short, Andrew D. Frugé

**Affiliations:** 1 Department of Nutritional Science, Auburn University, Auburn, AL 36849, USA; 2 College of Nursing, Auburn University, Auburn, AL 36849, USA

**Keywords:** nursing students, sleep quality, diet quality, anxiety, physical activity, interoception

## Abstract

Baccalaureate nursing students are at increased risk for anxiety and related mood disorders. We conducted a cross-sectional study to explore the relationships among anxiety symptoms measured by the Generalized Anxiety Disorder (GAD-7) questionnaire and lifestyle behaviors including habitual diet, sleep quality (Pittsburg Sleep Quality Index [PSQI]), and physical activity. Descriptive statistics were obtained for sample characteristics, and Pearson correlations and backward stepwise linear regression explored relationships between the GAD-7 scores, the Multidimensional Assessment of Interoceptive Awareness, version 2 (MAIA-2) subscales, and other variables. Sixty-eight students completed the survey, with 38% having moderate-to-severe anxiety. On average, respondents had moderate diet quality (Healthy Eating Index median 60/100 [range 51–75]), had high sleep quality (PSQI median 7/21 [range 4–10]), and were highly active, with a median of 43 (range 24–78) weekly metabolic equivalent (MET) hours. Sixty-seven out of 68 respondents indicated a willingness to change lifestyle behaviors; the most prevalent time-related factors were school and social commitments, with stress and financial constraints being reported among half or more of respondents. Regression analysis determined that PSQI (β = 0.446) and the MAIA-2 Not-Worrying subscale (β = −0.366) were significant (*p* < 0.001 for both) predictors of anxiety severity. These results indicate that mindfulness and sleep hygiene may be the most actionable foci for interventions to reduce anxiety in baccalaureate nursing students. This study was not registered as a clinical trial.

## 1. Introduction

Anxiety disorders rank among the top ten leading causes of disability in adolescents and young adults, impacting approximately 301 million people globally [1,2]. Concurrently, psychological distress among American college students has reached unprecedented levels [3], with 61% of students reporting anxiety and depression as primary reasons for seeking counseling [4]. Amid a myriad of stress-inducing factors, eight in ten students report experiencing extreme stress regarding their uncertain future [3,5]. Baccalaureate nursing programs pose unique challenges as students must navigate rigorous and fast-paced coursework while consistently demonstrating robust critical thinking skills and effective time management [6]. The most prominent stressors for nursing students include academic demands, fear of making mistakes with patients or when handling technical equipment, relationships within the clinical environment, and the responsibilities associated with patient and family care [7,8]. The integration of theoretical knowledge and clinical practice in nursing education not only makes the process challenging and stressful but also leads to heightened anxiety, with studies indicating that over 30% of nursing students experience high levels of anxiety [9].

Several factors that predict academic stress and achievement are associated with life satisfaction, psychological well-being, one’s relationship with happiness, and one’s locus of control [10]. One study found that 66.5% of surveyed nursing students had experienced varying levels of anxiety crises, with 48.8% of these students choosing not to seek assistance for mitigation of their anxiety or stress [11]. Academic stress has been linked to health concerns such as physical exhaustion, sleeping disorders, irritability, negative thoughts, and heightened nervousness [12]. Additionally, increased stress levels have been observed to contribute to the consumption of unhealthy foods while simultaneously reducing the intake of healthy options [13]. College students experiencing higher stress levels demonstrated a greater tendency to dine out, indulge in sugary snacks, and skip meals, ultimately leading to overeating [14,15]. One study involving 523 students revealed that nearly 83% experienced moderate-to-high levels of stress, while over 80% exhibited low-to-medium levels of self-regulation in their eating habits [15]. Emotional eating and overeating have been linked to increased stress, depression, and increased body mass index (BMI) [16]. In contrast, regular physical activity and increased sleep have a positive influence on stress markers in adults [17,18].

Given the greater demands and potential stressors of nursing school and increased awareness of anxiety in undergraduate students, we sought to assess anxiety symptoms, diet quality, physical activity, sleep quality, and interoceptive awareness among undergraduate nursing students. Additionally, we aimed to explore the relationships among these factors to determine predictors of increased anxiety. We hypothesized that the majority of nursing students would report experiencing mild-to-moderate anxiety levels and exhibit lower interoceptive awareness.

## 2. Materials and Methods

### 2.1. Participants

A convenience sample was obtained after exempt Institutional Review Board (IRB) approval was provided by the Auburn University IRB. Students in the second and third semester of the baccalaureate nursing program were approached during the first week of their respective semesters. An email with an information letter and survey link was provided on 18 August 2023, and the Qualtrics survey was open a total of ten days, with additional reminders sent on days three and seven. Surveys were completed anonymously; however, students could opt in to a draw for one of 25 10USD gift cards via a separate 

### 2.2. Measures

#### 2.2.1. Generalized Anxiety

The seven-item generalized anxiety disorder screener (GAD-7) was used to assess anxiety symptom severity [19]. Each item is rated on a four-point Likert scale (0–3), with total scores ranging from 0 to 21, where higher scores indicate greater severity of anxiety. The GAD-7 has shown good reliability and construct validity with a Cronbach alpha ranging from α = 0.88 to α = 0.93 [19,20].

#### 2.2.2. Interoceptive Awareness

Interoceptive awareness was assessed using the Multidimensional Assessment of Interoceptive Awareness, version 2 (MAIA-2). The MAIA-2 consists of a 37-item questionnaire measuring across eight subscales: (i) Noticing, which assesses awareness of various body sensations; (ii) Not-Distracting, which evaluates the tendency to avoid ignoring sensations of pain or discomfort; (iii) Not-Worrying, related to avoiding emotional distress in response to pain; (iv) Attention Regulation, concerning the control and maintenance of attention to body sensations; (v) Emotional Awareness, which links body sensations to emotional states; (vi) Self-Regulation, involving the management of psychological distress through body awareness; (vii) Body Listening, which focuses on gaining insights by listening to the body; and (viii) Trusting, the perception of the body as safe and reliable. The MAIA-2 employs a 6-point Likert scale (0–5) for scoring, with average scores for each subscale indicating the level of interoceptive awareness, where higher scores represent greater awareness. The subscales exhibit Cronbach’s alpha values ranging from 0.64 to 0.83, indicating their reliability [21].

#### 2.2.3. Sleep Quality

Sleep quality was assessed using the Pittsburg Sleep Quality Index. The 19-item self-report questionnaire measures seven components of sleep: subjective sleep quality, sleep latency, sleep duration, habitual sleep efficiency, sleep disturbances, use of sleeping medication, and daytime dysfunction. A score above 5 on this scale effectively distinguishes between good and poor sleepers. The Global Sleep Quality scale, with a reported Cronbach’s alpha of 0.83, demonstrates its reliability [22].

#### 2.2.4. Physical Activity

The modified 5-item physical activity questionnaire (PAQ-M) assesses the number of hours spent weekly in physical activity across light, moderate, and vigorous activities as well as in resistance training. The PAQ-M has test–retest reliability with an intraclass correlation of 0.87 [23]. Times spent in each domain were multiplied by an average metabolic equivalent (MET) to estimate total MET hours per week of physical activity [24].

#### 2.2.5. Diet Quality

Seven questions were used to assess habitual diet over the previous thirty days using the Global Assessment Tool 2.0 and Health Eating Score-5 (HES-5) [25]. A Cronbach test determined that HES-5 yielded an internal consistency reliability coefficient of 0.81 [26]. The short Healthy Eating Index (s-HEI) scoring criteria were used to scale responses to the Dietary Guidelines for Americans HEI, resulting in a relative diet quality score out of 100 [27].

#### 2.2.6. Perceived Health and Willingness to Change

Perceived health was assessed with the first question from the RAND Short-Form 12-Item Health Survey [28], and four questions asked whether a change in health behaviors was currently a priority, the timing of willingness to change, and barriers to change [29]. In the general US population, the RAND Short-Form 12-Item Health Survey demonstrated reliability with test–retest correlations of 0.89 for the 12-item Physical Component Summary and 0.76 for the 12-item Mental Component Summary [28].

#### 2.2.7. Demographics and Anthropometrics

Demographic variables were obtained using standard US census methods. Current height was reported in feet and inches, and current weight was reported in pounds. BMI was calculated and reported in kg/m^2^.

### 2.3. Statistical Analysis

Statistical analyses were conducted in SPSS Version 29.0 (IBM Corp, Armonk, NY, USA). Descriptive statistics were obtained for sample characteristics, and Pearson correlations explored relationships between independent variables and GAD-7 scores and MAIA-2 subscales. Backward stepwise linear regression with GAD-7 score as the dependent variable was used to determine the predictive value of highly correlated factors.

## 3. Results

Of 180 potential second- and third-semester students, 68 (37.7%) completed the survey. Demographics characteristics were mostly representative of the nursing student population with regard to gender, race, and ethnicity (Table 1). Physical activity levels were skewed, ranging from 24 to 48 MET hours/week. Diet quality was moderate overall, ranging from 51 to 75 out of a possible 100 points. Sleep quality was concentrated on the low (better) end, ranging from 4 to 10 out of 21 points. Finally, most students had normal-weight BMI. According to GAD-7 scores, roughly 60% of respondents had minimal or mild anxiety, with 25% having moderate and 15% having severe anxiety. No students self-reported poor health, while slightly more than half of students reported very good or excellent health.

**Figure 1:**
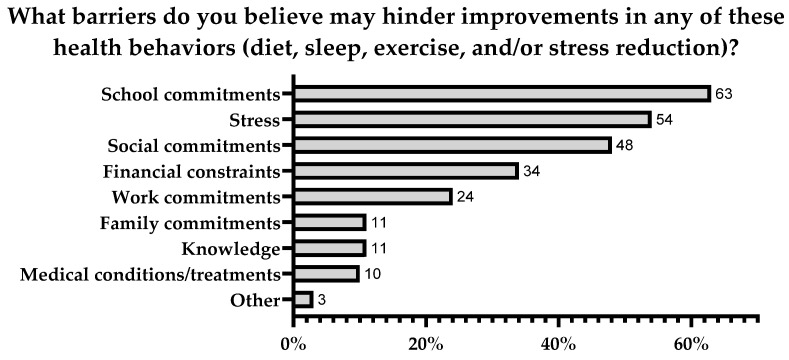
Self-reported perceived barriers to changing health behaviors in baccalaureate nursing students (*n* = 68).

Sixty-seven out of 68 students reported they were willing to change their health behaviors, providing one to eight specific barriers, with four as the median. The most prevalent time-related factors were school and social commitments, with stress and financial constraints rounding out the other top choices reported by at least half of the students (Figure 1).

Backward stepwise regression, with GAD-7 scores as the dependent variable, reduced five highly correlated independent variables to three, omitting barriers to change and the MAIA-2 Trusting subscale in the final model. Sleep quality (β = 0.446) and the MAIA-2 Not-Worrying subscale (β = −0.366) were the remaining significant (*p* < 0.001 for both) predictors of anxiety severity (Table 2).

**Figure 2:**
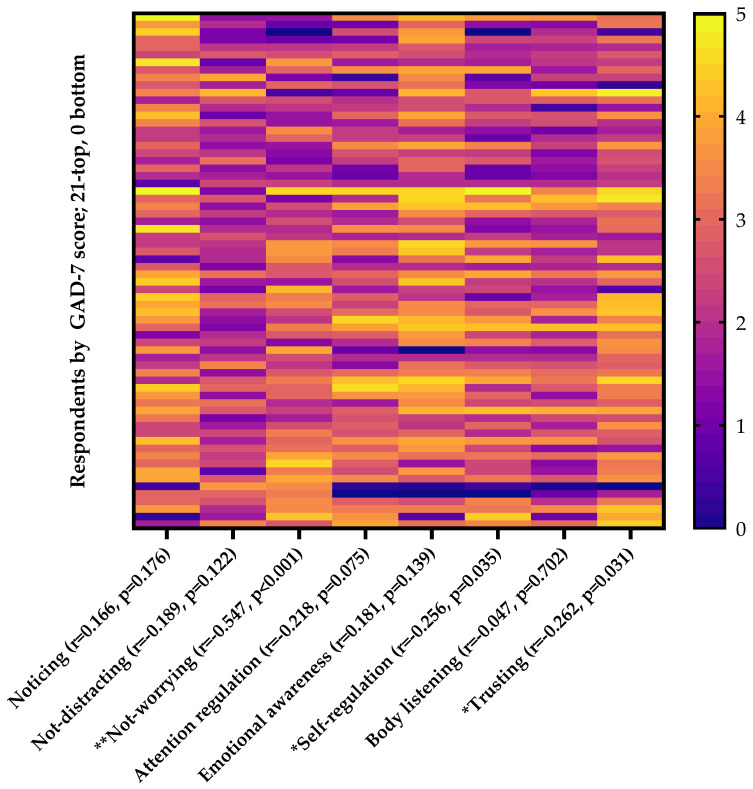
Heatmap of MAIA-2 subscales ordered by the respondents’ GAD-7 score. Rows represent individual respondents’ subscale scores, with respondents (*n* = 68) listed by decreasing GAD-7 scores from top to bottom. * *p* < 0.05; ** *p* < 0.005.

Correlations between GAD-7 score and MAIA-2 subscales are displayed in Figure 2. Anxiety symptom severity was most strongly associated with the Not-Worrying subscale (r = −0.547, *p* < 0.001), followed by the Trusting (r = −0.262, *p* = 0.031), Self-Regulation (r = −0.256, *p* = 0.035), and Attention Regulation (r = −0.218, *p* = 0.075) subscales.

## 4. Discussion

In our study of baccalaureate nursing students, we found that respondents generally reported moderate diet quality and high sleep quality and maintained high levels of physical activity. However, GAD-7 testing showed that 40% of students experienced moderate-to-severe anxiety. Notably, sleep quality and the Not-Worrying subscale of the MAIA-2 questionnaire emerged as significant predictors of anxiety severity. Our analysis revealed that various MAIA-2 subscales, particularly Not-Worrying, Trusting, Self-Regulation, and Attention Regulation, exhibited the strongest correlations with anxiety symptom severity. Importantly, 99% of respondents reported that they were willing to change their health behaviors for the better.

Given that a majority of respondents self-reported minimal or mild anxiety levels along with moderate-to-high diet quality, physical activity, and sleep quality, our findings are consistent with previous research exploring the connections between health behaviors and mental health [6,17,18]. A systematic review conducted by Saha and colleagues [30] highlights the relationship between diet and symptoms of anxiety and depression in college students. Among the 21 studies reviewed, including four interventional studies, the majority demonstrated positive associations between a healthy diet and improved mental health during college. In a similar vein, a series of pilot studies investigated the impact of regular physical activity on mental health and well-being, as well as the health benefits of short-term aerobic exercise, in university students. These studies revealed a negative correlation between regular exercise and self-reported anxiety, depression, and perceived psychosomatic stress. Additionally, cardiovascular fitness was inversely associated with self-reported anxiety, depression, and stress related to uncertainty [31]. A cross-sectional study in Indian college students observed significant inverse relationships between moderate and high physical activity levels and anxiety and depression scores, while sleep quality was positively associated with anxiety and depression [32]. Furthermore, in a moderation mediation analysis conducted on higher education students from seven countries, it was found that perceived stress and anxiety were negatively associated with sleep quality, but this relationship was weakened by increased psychological resilience [33]. Collectively, these findings highlight the effects of lifestyle behaviors on reducing anxiety levels in college students.

Interoceptive awareness, as defined by Price and Hooven [34], refers to the capacity to recognize internal bodily sensations, encompassing heart rate, respiration, hunger, satiety, temperature, pain, and emotional sensations. Several studies have investigated the relationships between anxiety and interoception in this population. Our study found that the Not-Worrying, Trusting, and Self-Regulation subscales from the MAIA-2 displayed strong negative correlations with anxiety levels among nursing students. Other studies of college students have revealed that higher interoceptive scores are associated with increased feelings of self-efficacy in learning, improved self-regulation of attention, enhanced academic performance, and higher grade point averages [35,36]. Interoceptive awareness also serves as an effective measure for assessing the outcomes of stress and relaxation training in practicing nurses. One study found that Self-Regulation score was negatively correlated with and predictive of anxiety in both hospital nurses and university students, while nurses specifically exhibited higher anxiety levels and lower mean Body Listening scores compared to university students [37]. Similar to our findings, Kabir et al. found that MAIA-2 subscales Noticing, Not-Distracting, Not-Worrying, Emotional Awareness, Self-Regulation, and Trusting negatively predicted anxiety in both groups.

Despite the fact that most participants self-reported having very good or excellent health, a significant majority of them expressed a willingness to change their health behaviors. Participants identified school and social commitments, stress, and financial constraints as the most relevant perceived barriers to making changes in their health behaviors. While certain barriers may be non-modifiable (e.g., financial constraints and medical conditions), addressing stress and time management offers potential solutions to these challenges. In one trial, progressive muscle relaxation exercises performed for 45 min 5 days per week for two weeks, along with an additional 10–15 min training session performed every day at home, reduced anxiety among nursing students [38]. Similarly, a study conducted by Almarcha and colleagues found that participants involved in an exercise regime experienced increased mental health and interoceptive awareness scores. Interoceptive scores increased further for those who were able to co-design their exercise regime [39]. Finally, in a study assessing the impact of a time management training program on time management and anxiety among nursing undergraduate students, significant improvements were reported. Students paid more attention to the value of time, engaged in more time management behaviors, and increased their time efficacy [40].

Recent research offers strong evidence for the efficacy of targeted interventions in reducing stress and anxiety among nursing students and healthcare providers. For example, a systematic review identified 18 studies highlighting cognitive behavioral therapy (CBT) and mindfulness-based stress reduction (MBSR) as particularly effective in alleviating stress, anxiety, and depression among undergraduate nursing students [41]. Additionally, a specialized two-phase intervention program incorporating CBT and progressive muscle relaxation demonstrated significant reductions in stress and anxiety levels among nursing students after just the first three-month phase, enhancing their ability to manage clinical demands more effectively [42]. Moreover, Heeter and colleagues found that engaging in 10–12 min of yoga and meditation via smartphone apps over six weeks significantly enhanced participants’ perceived ability to focus on body sensations and emotions. Such technology-assisted meditation programs have also been shown to enhance interoceptive awareness and reduce compassion fatigue and burnout among healthcare providers [43]. Building on these findings, future interventions could integrate cognitive behavioral strategies, reinforced peer support, and innovative technology-assisted mindfulness practices to comprehensively address the mental health needs and enhance the overall well-being of nursing students.

This study possesses noteworthy limitations that impact the generalizability of our findings. First, we acknowledge the subjective nature of our data, which is inherent in survey research. While we used validated assessments of dietary, physical activity, and sleep habits, recall bias is highly probable. Second, the sample was predominantly composed of white females. This lack of diversity makes it challenging to extrapolate the study’s findings to a broader population, though it is representative of most baccalaureate nursing programs in the United States. Additionally, the administration of the survey exclusively in a southeastern United States university further limits its applicability to the national student population or associate degree nursing programs.

## 5. Conclusions

These results suggest that anxiety is highly prevalent among undergraduate nursing students, and mindfulness and sleep hygiene may be ideal behaviors to modify for anxiety reduction. Baccalaureate nursing programs are encouraged to assess and intervene to promote programmatic and long-term professional success for its current and future nursing students.

## Figures and Tables

**Table 1:** Characteristics of baccalaureate nursing student participants.

	*N* (%)
Female gender	64 (94.1%)
White race	66 (97.1%)
Hispanic ethnicity	2 (2.9%)
Age; median (range)	21 (19–26)
Earned associate degree	2 (2.9%)
Prior military service	2 (2.9%)
Lifestyle factors	
MET hours/week; median (range)	43 (24–78)
Diet Quality (HEI Score); median (range)	60 (51–75)
Sleep Score (PSQI Global Score); median (range)	7 (4–10)
BMI; mean (SD)	23.2 (3.8)
GAD-7	
Minimal anxiety	25 (36.8%)
Mild anxiety	16 (23.5%)
Moderate anxiety	17 (25%)
Severe anxiety	10 (14.7%)
General health status	
Fair or good	31 (45.6%)
Very good or excellent	37 (54.4%)
Willing to change	67 (98.5%)
Barriers to change; median (range)	4 (1–8)

**Table 2:** Backward stepwise regression of variables predicting anxiety severity in baccalaureate nursing students (*n* = 68).

Model	Dependent Variable: GAD-7	Beta	t	Sig.
	(Constant)		3.135	0.003
	Sleep quality (PSQI)	0.413	4.296	<0.001
	Perceived health	−0.159	−1.715	0.091
	Number of barriers to behavior change	0.128	1.450	0.152
	MAIA Not-Worrying	−0.361	−3.868	<0.001
	MAIA Trusting	−0.045	−0.477	0.635
2	(Constant)		3.295	<0.001
	Sleep quality (PSQI)	0.425	4.602	<0.001
	Perceived health	−0.173	−1.954	0.055
	Number of barriers to behavior change	0.133	1.526	0.132
	MAIA Not-Worrying	−0.357	−3.865	<0.001
3	(Constant)		4.679	<0.001
	Sleep quality (PSQI)	0.446	4.823	<0.001
	Perceived health	−0.168	−1.887	0.064
	MAIA Not-Worrying	−0.366	−3.93	<0.001

## Data Availability

Data are available upon reasonable request to the corresponding author.

## Public Involvement Statement

No public involvement in any aspect of this research.

## Guidelines and Standards Statement

This manuscript was drafted against the STROBE guidelines for cross-sectional studies.
